# Searching for DNA Damage: Insights From Single Molecule Analysis

**DOI:** 10.3389/fmolb.2021.772877

**Published:** 2021-11-05

**Authors:** Matthew A. Schaich, Bennett Van Houten

**Affiliations:** ^1^ UPMC Hillman Cancer Center, University of Pittsburgh, Pittsburgh, PA, United States; ^2^ Department of Pharmacology and Chemical Biology, School of Medicine, University of Pittsburgh, Pittsburgh, PA, United States; ^3^ Molecular Biophysics and Structural Biology Graduate Program, University of Pittsburgh, Pittsburgh, PA, United States

**Keywords:** single molecule fluorescence microscopy, DNA tightrope, nucleotide excision repair, base excision repair, DNA damage

## Abstract

DNA is under constant threat of damage from a variety of chemical and physical insults, such as ultraviolet rays produced by sunlight and reactive oxygen species produced during respiration or inflammation. Because damaged DNA, if not repaired, can lead to mutations or cell death, multiple DNA repair pathways have evolved to maintain genome stability. Two repair pathways, nucleotide excision repair (NER) and base excision repair (BER), must sift through large segments of nondamaged nucleotides to detect and remove rare base modifications. Many BER and NER proteins share a common base-flipping mechanism for the detection of modified bases. However, the exact mechanisms by which these repair proteins detect their damaged substrates in the context of cellular chromatin remains unclear. The latest generation of single-molecule techniques, including the DNA tightrope assay, atomic force microscopy, and real-time imaging in cells, now allows for nearly direct visualization of the damage search and detection processes. This review describes several mechanistic commonalities for damage detection that were discovered with these techniques, including a combination of 3-dimensional and linear diffusion for surveying damaged sites within long stretches of DNA. We also discuss important findings that DNA repair proteins within and between pathways cooperate to detect damage. Finally, future technical developments and single-molecule studies are described which will contribute to the growing mechanistic understanding of DNA damage detection.

## Introduction

The human genome is ceaselessly showered by various DNA-damaging agents. These can include endogenous agents, such as reactive oxygen species created as a byproduct of respiration, or exogenous species, including environmental factors like sunlight or air pollution (Hakem, 2008). The levels of DNA damage can greatly vary depending on a cell’s environment, but it has been estimated that as many as 70,000 lesions of DNA damage are generated per cell per day (Tubbs and Nussenzweig, 2017). DNA lesions exhibit varied chemistry and alter the structure of DNA in numerous ways. Some helix distorting lesions such as UV-photoproducts inhibit transcription or block DNA replication, while other lesions such as 8-oxoG have high mutagenic potential. DNA damage induced-signaling can be protective by inhibiting the cell cycle to facilitate repair or catastrophic by initiating programmed cell death (Roos and Kaina, 2006; Peters and Gonzalez, 2018).

Fortunately, numerous DNA repair pathways have evolved to combat DNA damage and promote genome stability. In each pathway, DNA repair proteins must scour the genome for DNA damage, correctly identify a chemically-altered base, and finally replace the offending nucleotide with the original sequence. This review focuses on our structural knowledge of how proteins involved in base excision repair (BER) and nucleotide excision repair (NER) recognize lesions in the DNA, and how single molecule techniques have enhanced our understanding of the dynamics of these processes.

### Barriers Facing the Detection of DNA Damage

The detection of DNA damage embedded in a long chain of undamaged nucleotides presents a difficult challenge, as DNA repair proteins that detect altered bases also have non-specific DNA binding affinity. Thus, the vast excess of nondamaged DNA in a cell acts a competitive inhibitor. In a biological context, the competitor nondamaged nucleotide is four-six orders of magnitude more abundant than a DNA repair protein’s true substrate. Because inhibitor concentration greatly exceeds that of the substrate, repair proteins must rapidly search for sites of damage while simultaneously binding with high stability for the damage once it is encountered. This speed-stability paradox first proposed by Slutsky and Mirny (2004) for specific protein recognition of DNA sequences seems to be a special case for damage recognition proteins (Slutsky and Mirny, 2004): their intricate dance with DNA helps to sculpt the damage into a higher affinity binding site through conformational proof-reading (Ghodke et al., 2014). Furthermore, the search for DNA damage can be inhibited by presence of other DNA binding proteins, including nucleosomes and transcription factors, which further decreases the likelihood of repair (Meas et al., 2019). Facing the challenges of difficult identification, abundance of competitor DNA, and lack of chromatin accessibility, it is nothing short of incredible that DNA repair pathways efficiently find and repair their substrates. Understanding how DNA repair proteins search the entire cell nucleus for damaged sites is one of the fundamental problems in the field of genome stability.

### Overview of NER and BER Pathways

**FIGURE 1:**
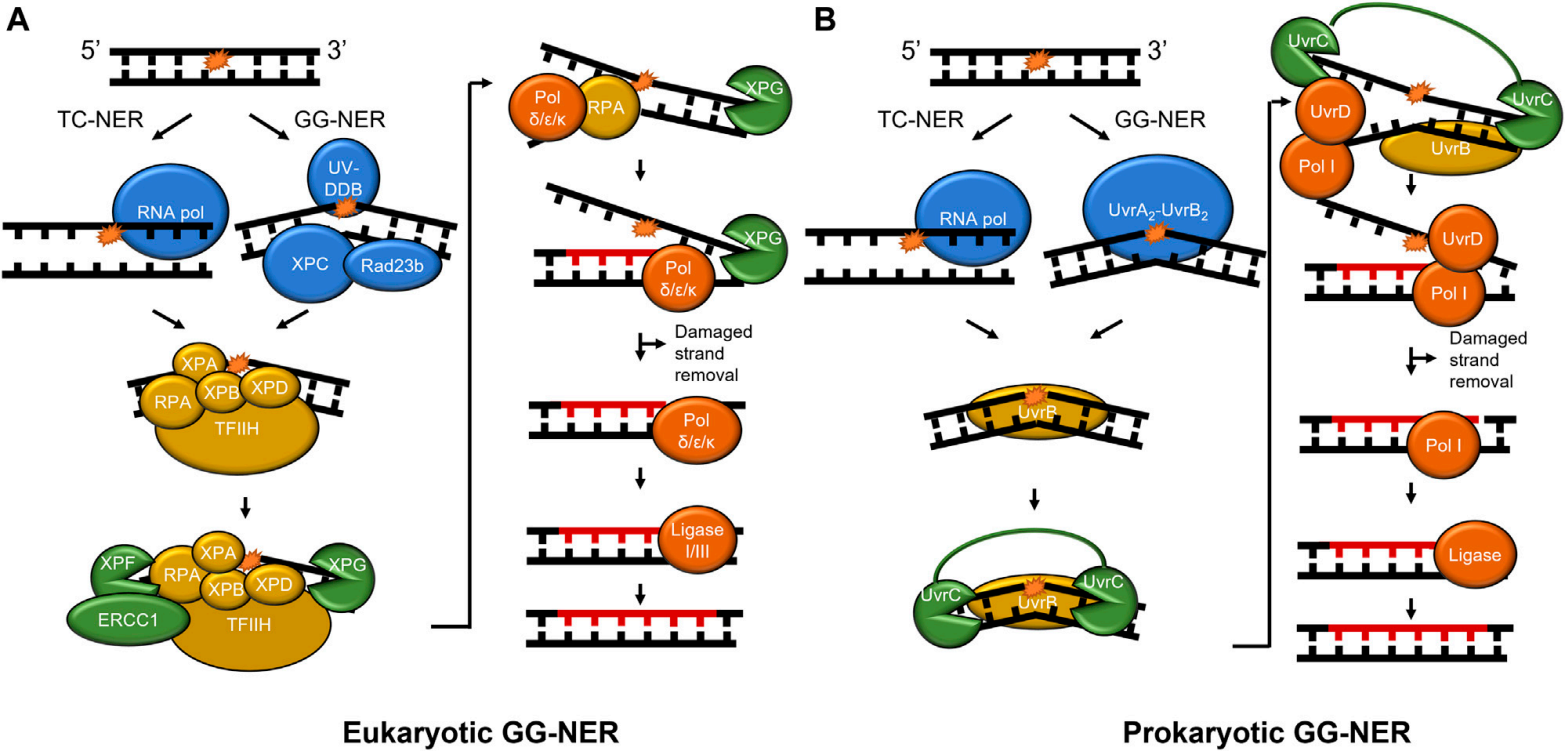
NER pathways in mammals and prokaryotes. **(A)** The mammalian NER pathway. Damage is recognized by UV-DDB and XPC-Rad23b (blue) in global-genome NER (GG-NER). Stalling of RNA polymerase (blue) initiates transcription-coupled NER (TC-NER). Then, damage verification is performed by TFIIH, XPA, RPA, ERCC1, XPB, and XPD (gold). XPF incises on the 5′ side of the damage and XPG incises on the 3′ side following DNA synthesis (both shown in green). Lastly, the repair is performed by DNA polymerase δ/ε/κ and sealed by ligase (red). **(B)** Prokaryotic NER is performed by UvrA, UvrB, UvrC, UvrD, Pol I, and ligase. As in mammalian NER, RNA polymerase stalling initiates TC-NER, but GG-NER is initiated by UvrA_2_-UvrB_2_. For simplicity, CSA, CSB, CSA, XAB2, and HMGN1 are not shown in mammalian TC-NER, and Mfd not shown in bacterial TC-NER. A 24–32 nucleotide region **(A)** and 12–13 nucleotide region **(B)** is replaced in each pathway (not to scale). To show functional overlap across the pathways, proteins are color-coded with damage detection in blue, verification in gold, incision in green, and repair in red.

Nucleotide excision repair undergoes a multistep pathway to successfully detect and remove DNA lesions, particularly those caused by UV damage such as cyclobutane pyrimidine dimers (CPD) and 6-4 photoproduct lesions (Figure 1). NER functions in all three branches of life from prokaryotes to mammalian cells, proceeding in four general steps: damage recognition, damage verification, damage removal, and repair synthesis. Damage recognition proceeds through one of two subpathways, global-genome NER (GG-NER) or transcription-coupled NER (TC-NER). In GG-NER, damage recognition is canonically carried out by UvrA and UvrB in prokaryotic cells and UV-DDB and XPC-RAD23 B-CENT2 in mammalian cells (Camenisch and Nageli, 2008; Kad et al., 2010; Kisker et al., 2013). In TC-NER, the lesion stalls transcription to initiate the pathway (Scharer, 2013). In bacteria, the Mfd protein helps displace the stalled RNA polymerase, while in mammalian systems the dual action of CSA and CSB are necessary for this function (Strick and Portman, 2019; Kraithong et al., 2021; van den Heuvel et al., 2021). After damage recognition, the TFIIH (transcription factor IIH) complex is recruited to the site of the lesions to allow XPD to verify the presence of DNA damage, along with XPA and RPA to stabilize the verification complex. In contrast, verification is performed by UvrB in prokaryotic cells followed with DNA incisions by UvrC on both the 5′ and 3′ sides of the lesion to facilitate damage removal (Hughes et al., 2013). The UvrBC-post-incision complex and the damaged containing oligonucleotide are released by the dual action of UvrD and DNA polymerase I (Pol I). The resulting repair patch is sealed with DNA ligase (Kisker et al., 2013; Lee et al., 2013). In the mammalian system, the heterodimer XPF/ERCC1 nicks the DNA on the 5′ side of the lesion, a DNA polymerase fills in the nicked strand, and in a coupled manner XPG nicks the undamaged DNA on the 3’ side of the lesion. To finish the repair, DNA ligase I or III will be recruited to seal the nick, replacing the lesion with new, undamaged, DNA (Scharer, 2013).

**FIGURE 2:**
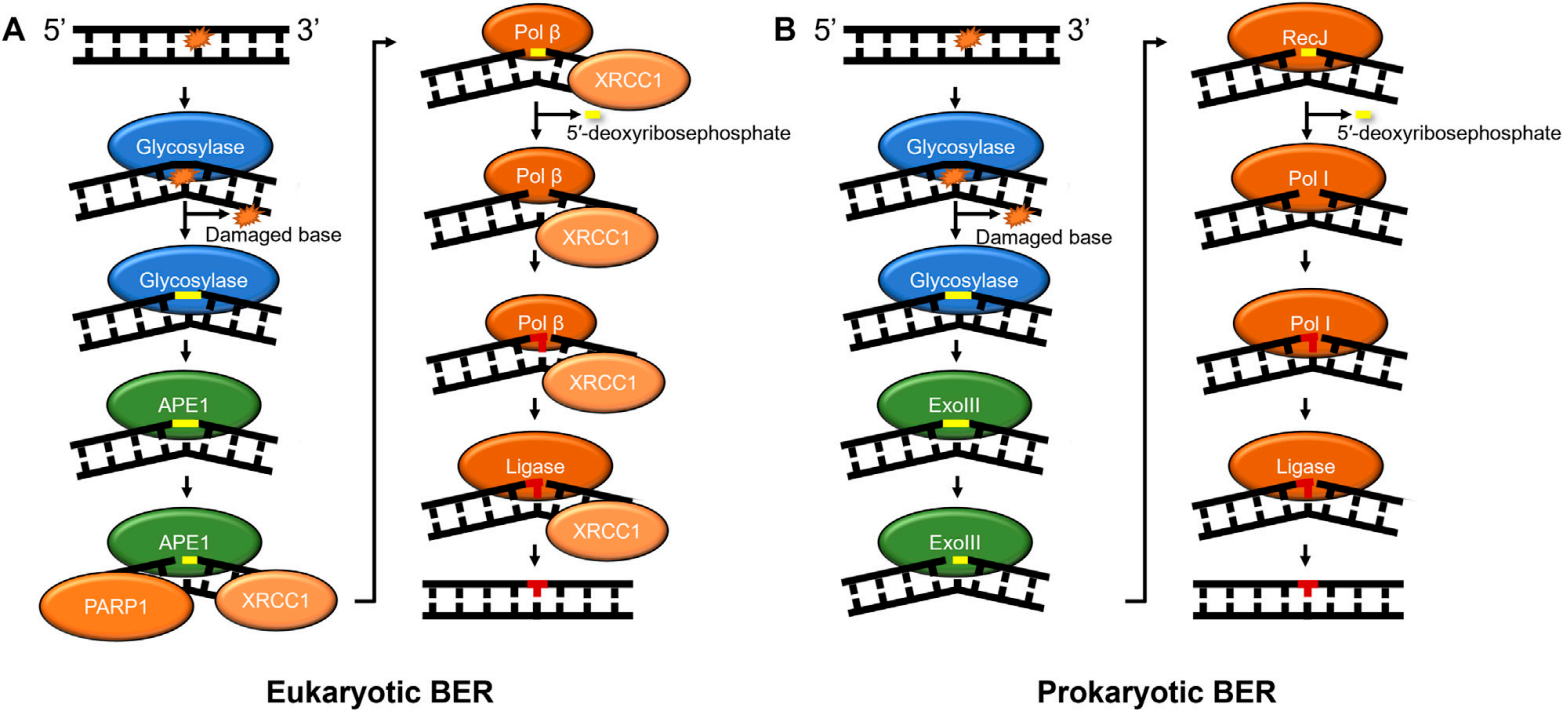
BER pathways in mammals and prokaryotes. **(A)** Mammalian base excision repair is initiated by a lesion specific glycosylase (monofunctional shown). Subsequently, APE1 further processes the glycosylase product, nicking the phosphate backbone on the 5′ side of abasic site. Then, the DNA is processed by the lyase activity of Pol β to remove the 5′-deoxyribosephosphate and the polymerase activity of Pol β regenerates the undamaged sequence which is sealed by DNA ligase. **(B)** Prokaryotic BER proceeds with similar intermediates to mammalian BER, with some prokaryotes using exonuclease III (XthA) to perform the AP-endonuclease reaction, RecJ to perform the lyase reaction, and Pol I inserting undamaged nucleotides to replace the damage. Some bi-functional glycosylases, such as Nth or Endo III, with associated lyase activities produce beta-elimination generating a 3′OH and a deoxyribose moiety on the 5′ side that needs to be further processed (not shown). Other bi-functional glycosylases like Endo VIII, or NEIL1-3 have associated or beta-delta elimination removing the entire base leaving a one base gap (not shown). As in Figure 1, proteins are color-coded with damage detection in blue, verification in gold, incision in green, and repair in red.

BER in prokaryotes and eukaryotes also acts as a primary responder for a diverse array of damages, including those caused by oxidative stress, such as 8-oxoguanine and 2,6-diamino-4-oxo-5-formamidopyrimidine (FapyG). Furthermore, BER enzymes remove damages caused by alkylative damage such as 1,N6-ethenoadenine (εA), deamination products of cytidine (resulting in guanine:uracil mispairs), and lesions such as spiroimino-dihydantoin and abasic sites (Whitaker et al., 2017). How are so many chemically diverse lesions identified? In humans, the search process is carried out by at least 11 different DNA glycosylases that each search for their own subset of DNA lesions (Kumar et al., 2020). After these glycosylases detect their cognate lesion substrate through a base-flipping mechanism, one class of glycosylases known as monofunctional cleaves the N-glycosidic bond between the base lesion and the deoxyribose sugar, whereas another class of glycosylase called bifunctional performs a different reaction in which they remove the modified base and nick the phosphate backbone of the DNA. Regardless of the glycosylase type, further processing of its product is required, which may include an apurinic/apyrimidinic endonuclease 1 (APE1 in humans) to bind the abasic sites and cleave the phosphate backbone or polynucleotide kinase (PNK) to process DNA backbone nicks created by glycosylases (Figure 2). The nick is further processed with the 8 kDa lyase activity of DNA polymerase β (Pol β) in mammals or RecJ in some prokaryotes (Dianov and Lindahl, 1994; Cheng et al., 2020). A DNA polymerase fills in the gap (Pol *ß* in mammals) and DNA ligase seals the nick after the undamaged base is inserted. In a subset of BER known as long-patch, the DNA polymerase inserts more than one nucleotide and creates a flap that is cleaved by FEN1 before ligation (Sattler et al., 2003). Furthermore, several scaffolding proteins are also involved throughout BER, including PARP1 and XRCC1 (Whitaker et al., 2017).

### Snapshots of NER and BER Proteins Detecting DNA Damage

**FIGURE 3:**
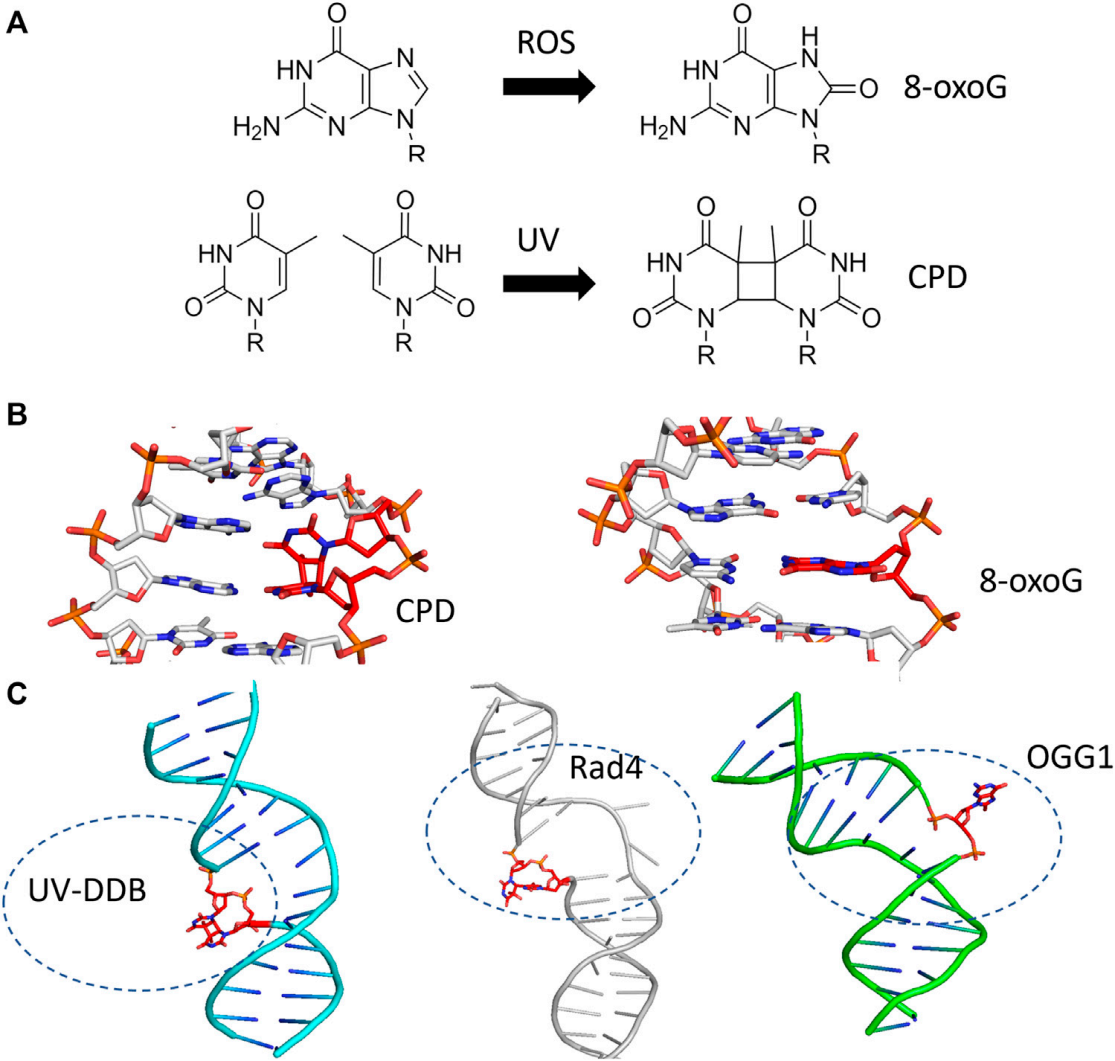
BER and NER substrates distort duplex DNA structure and are flipped out by damage detection proteins. **(A)** The formation of 8-oxoguanine by reactive oxygen species (ROS, top) and cyclobutane pyrimidine dimers by ultraviolet light (UV, bottom) generates substrates that largely resemble their undamaged counterparts. **(B)** The presence of 8-oxoG or CPD in duplex DNA mildly perturbs the structure of duplex DNA. Protein Data Bank (PDB) IDs: 183D and 1N4E used from (Lipscomb et al., 1995; Park et al., 2002) **(C)** When damage is bound by a damage detection protein, the structure exhibits DNA bending with the damage flipped outside of the DNA helix. Co-complexes are shown with the protein removed for UV-DDB binding CPD [cyan cartoon, PDB ID: 4A09 from (Fischer et al., 2011)], Rad4-Rad23 binding a 6-4 photoproduct [gray cartoon, PDB ID: 6CFI from (Paul et al., 2019)], and OGG1 binding 8-oxoG [green cartoon, from PDB ID:1EBM (Bruner et al., 2000)]. In each case, the DNA damage is indicated with red sticks.

The question of how DNA repair proteins recognize damage has been the subject of speculation since the discovery of the pathways (Hanawalt and Haynes, 1965; Hanawalt, 1993). 3D structures determined from X-ray crystallography of the BER glycosylases such as OGG1 (8-oxoguanine glycosylase) and UDG (Uracil DNA glycosylase) bound to their cognate lesions revealed models in which the glycosylases sharply bend the DNA at the damage site. This DNA bending helps facilitate the damaged base to flip outside of the DNA helix and into the active site pocket of the glycosylase (Figure 3) (Slupphaug et al., 1996; Bruner et al., 2000). Base flipping has also observed in the crystal structures of prokaryotic glycosylases, including MutY and MutM (formamidopyrimidine DNA glycosylase) (Fromme and Verdine, 2003; Fromme et al., 2004). Similarly, cryo-EM and crystal structures of proteins involved in the damage detection of NER, including UV-DDB and Rad4 (the yeast homolog of XPC), also revealed significant distortion of DNA secondary structure at the site of DNA damage. In both the UV-DDB and Rad4 structures, the DNA exhibited an even more extreme structural perturbation from canonical B-form, as two nucleotides are extrahelical in these structures compared to the single base flip of glycosylases (Figure 3) (Min and Pavletich, 2007; Scrima et al., 2008). These high-resolution structures provided insights for how different lesions, once encountered, are identified.

Contemporaneously with the determination of crystal structures of these DNA repair proteins, new techniques were being developed allowing researchers to view proteins working on DNA in real-time at single-molecule scale (Kabata et al., 1993; Erie et al., 1994; Hughes et al., 2014). In order to visualize single protein molecules, the fundamental challenge is to achieve conditions where the fluorescence of one molecule can be differentiated from the background signal. With purified proteins, single-molecule imaging conditions typically involve diluting samples down to low concentration (<10 nM) and selectively exciting a subset of the fluorophores through microscopy techniques such as oblique angle illumination or total internal reflection microscopy (TIRFM) (Ghodke et al., 2014; Fairlamb et al., 2021). In living cells, changing the volume to dilute proteins down to the levels needed for single-molecule imaging is not feasible. Therefore, super-resolution techniques including stochastic optical reconstruction microscopy (STORM), photoactivated localization microscopy (PALM), and highly inclined and laminated optical sheet (HILO) microscopy have been developed to allow single-particle tracking in live cells, as well as other imaging techniques (Betzig et al., 2006; Hess et al., 2006; Rust et al., 2006). For a comprehensive review on single-molecule techniques see here (Shashkova and Leake, 2017). In each of these cases, the fluorescence is depleted to increase the signal to noise ratio–this can be accomplished with selective excitation, limited photoactivation of fluorophores, or photobleaching most of the fluorophores prior to imaging. By utilizing techniques that allow single molecules to be imaged, a wealth of information can now be learned about how DNA repair proteins find their damaged substrates through 3-dimensional diffusion, their dwell times at lesion sites, the timing of how proteins factors assemble and disassemble during the repair process, and the heterogeneity in their populations.

**FIGURE 4:**
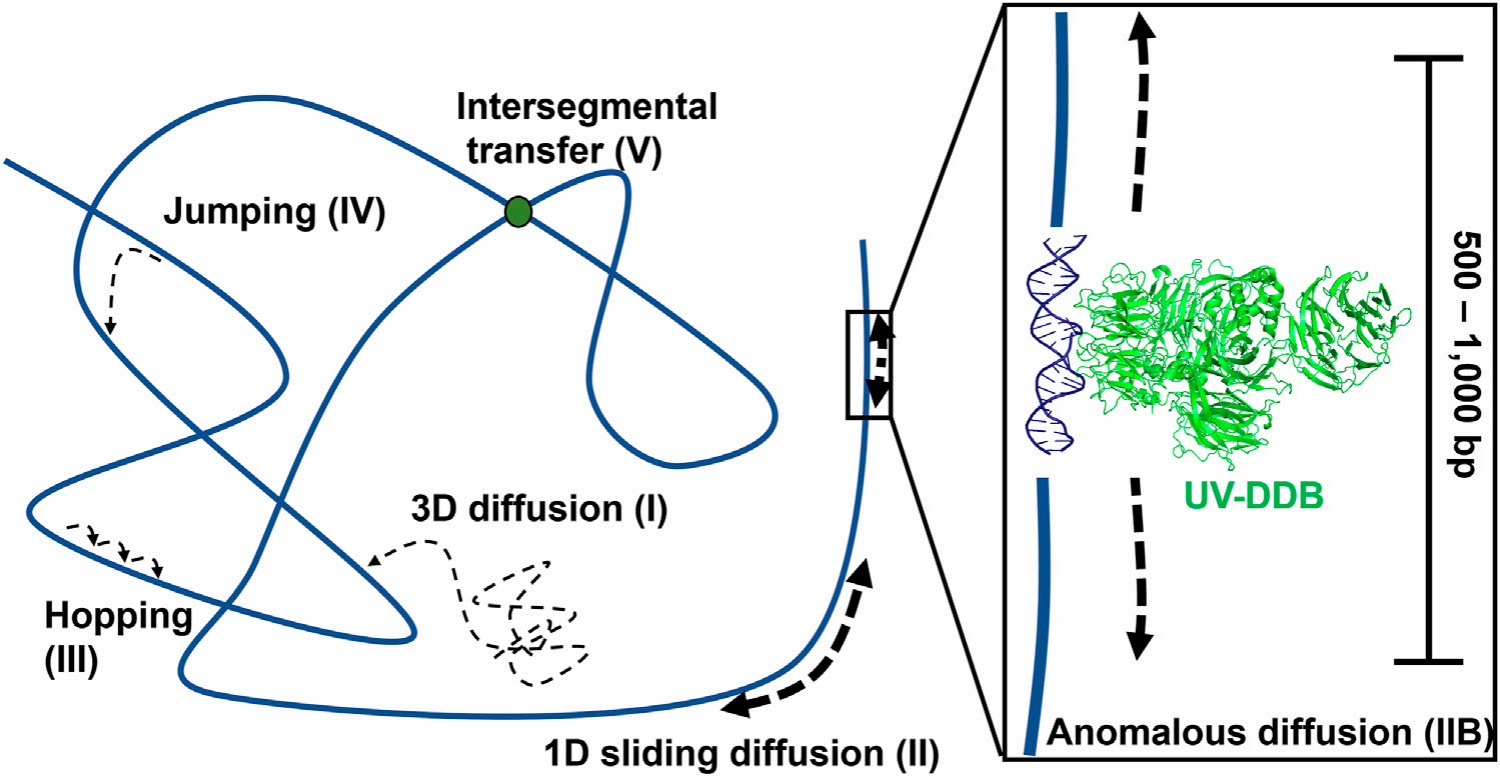
Modes of protein-DNA interaction. Types of protein motion shown include 3D diffusion (I), 1D linear diffusion/sliding (II), anomalous diffusion (IIB) a sub-diffusive movement along restricted regions of the DNA, hopping (III), jumping (IV), and intersegmental transfer (V). On the right side of the panel, a close-up view of anomalous diffusion is shown, in which a protein such as UV-DDB, in the presence of Mg^2+^, slides back and forth along 500–1,000 bp of DNA. PDB ID: 4E5Z (Yeh et al., 2012). DNA repair proteins probably use a combination of all search strategies to efficiently detect DNA damage.

Using these techniques, researchers were able to watch individual DNA repair proteins interrogate single strands of DNA duplexes for specific lesions (Blainey et al., 2006), and help answer the important question, how do DNA repair proteins efficiently search for DNA damage in a vast excess of undamaged DNA? Several potential mechanisms have been proposed (Figure 4 **)**. DNA repair proteins, if sufficiently high concentrations in the cell, can sample DNA for lesions using 3D diffusion, checking for damage during each encounter. Alternatively, DNA proteins can slide along the DNA through ionic interactions of positively charged amino acids with the negative charged phosphates. This 1D diffusion is driven by the motion of water molecules and results in a random walk in either direction. We have discovered that many DNA repair proteins including UV-DDB, Rad4, PARP1, UvrC, and XPA also display subdiffusive properties with limited motion along the DNA, which has been called anomalous diffusion (Kong et al., 2016; Kong and Van Houten, 2017; Liu et al., 2017; Cheon et al., 2019; Jang et al., 2019; Barnett et al., 2020; Beckwitt et al., 2020). With anomalous diffusion, the repair protein stays near the damaged site and does not slide large distances. Other DNA binding proteins or chromatin can obstruct long-distance sliding, so some proteins such as XPC and UvrBC have been found to form short dissociation and reassociation events called hopping (Hughes et al., 2013; Cheon et al., 2019). Hopping can be difficult to distinguish from sliding, but adding roadblocks on the DNA (as with XPC) or changing the ionic strength of solution (as with UvrBC) can allow for the differentiation between the two interaction modes. Prior to macro-dissociation proteins can rebind a section of DNA some distance away from the initial binding event; this is called jumping and has been observed with proteins like UvrA and UV-DDB. If the on and off rates are sufficiently rapid this would allow proteins efficiently search the genome. Finally, proteins consisting of multiple DNA binding motifs through oligomerization can bind to two DNA molecules simultaneously allowing rapid intersegmental transfer–this search mechanism is more challenging than the others to assay as it requires multiple strands of DNA, but has so far been observed on the mismatch repair protein MutLα (Gorman et al., 2012).

Below, we discuss how several key single-molecule studies further elucidated the mechanism by which NER and BER repair enzymes search for and detect DNA damage. We focus on the mechanisms of damage detection for both pathways, recent studies of crosstalk between the pathways, and finally overview future studies and technology under development may enable a better understanding of how DNA repair proteins detect DNA damage.

### Single-Molecule Studies of Bacterial NER Proteins

Single-molecule approaches such as DNA tightrope assays and atomic force microscopy (AFM) have played a prominent role in understanding how damage recognition occurs during NER in both bacterial and eukaryotic cells. Both of these approaches were reviewed by Kong et al. (Kong et al., 2017). The DNA tightrope assay consists of stringing up damaged DNA on poly-lysine coated 2–5 micron beads and visualizing quantum dot (Qdot) labeled proteins by oblique angle fluorescence microscopy. The latter tool, AFM, rasters a sharp tip (2–10 nm) attached to a flexible cantilever across atomically smooth mica surface to allow visualization of protein molecules bound to DNA in a label-free approach. As outlined above, bacterial NER is mediated by three key proteins UvrA, UvrB and UvrC. Structures of all three proteins (Karakas et al., 2007; Jaciuk et al., 2011; Pakotiprapha et al., 2012), combined with single molecule studies both with purified proteins and now in living cells has provided new insights into the function and dynamics of these proteins.

### Watching UvrA, UvrB, and UvrC Detect Damage in Real Time

**FIGURE 5:**
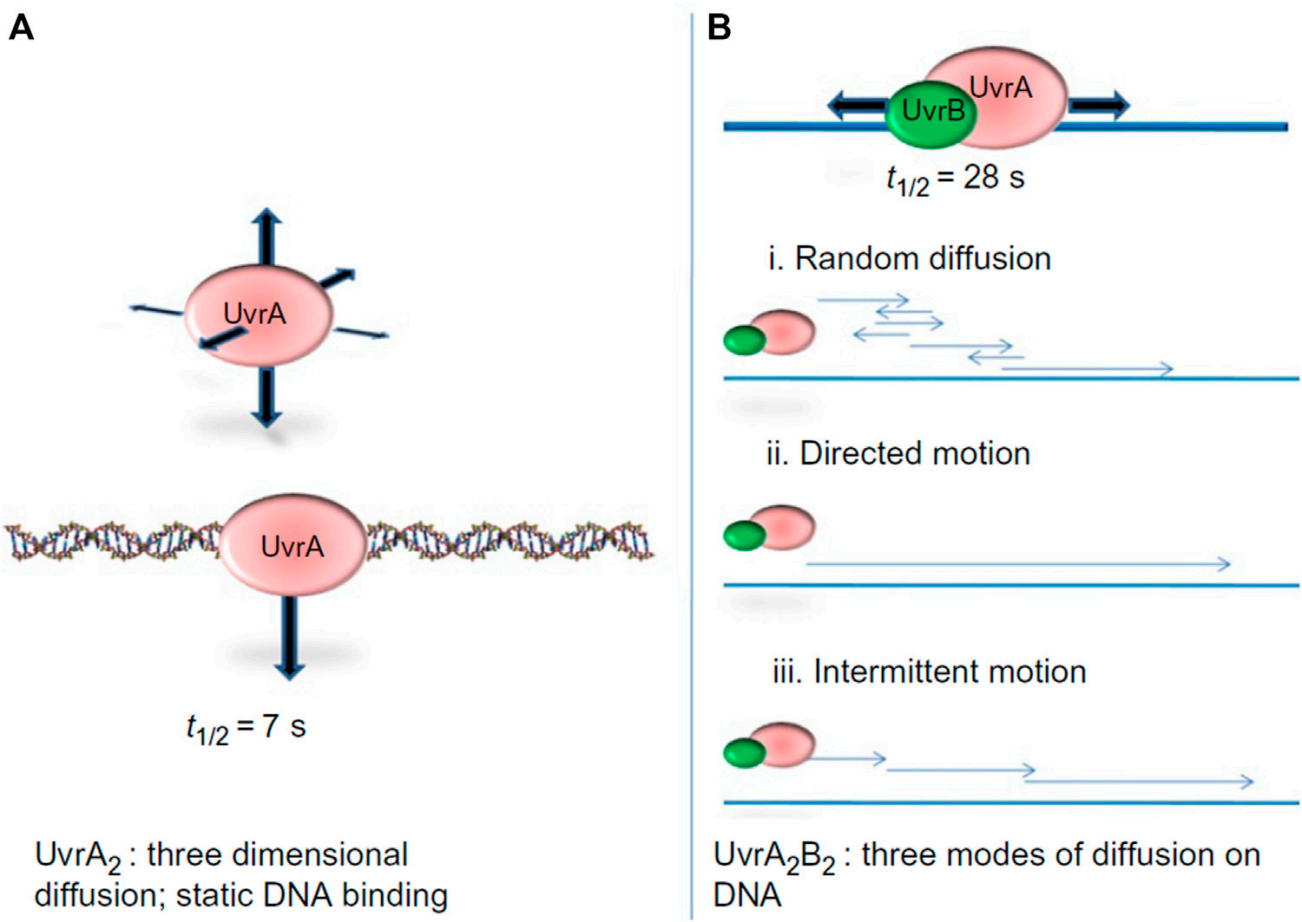
UvrA_2_ and UvrA_2_B_2_ complexes use different search strategies for finding damage. **(A)** Analysis of Qdot labeled UvrA showed that it performed a 3D-search and showed static binding of t_1/2_ of 7 s. **(B)** The UvrAB complex showed complex search behaviors on the DNA, including random linear diffusion, directed motion in the presence of ATP, and intermittent motion where the complex would pause sliding on the DNA for brief intervals. By combining a 3D search down with a1D search, UvrAB molecules are capable of efficiently searching the entire *E. coli* genome. Figure adapted with permission from (Kad and Van Houten, 2012).

The first study to watch bacterial UvrA and UvrB interact with DNA at the single molecule level demonstrated that these two proteins exhibit distinct DNA damage searching modes. In a wonderful collaboration with Neil Kad and David Warshaw, using oblique-angle fluorescence microscopy to study Qdot-labeled UvrA and UvrB on DNA tightropes, we observed that UvrA_2_ does an apparent three dimensional (3D) search in solution until it lands stably on DNA with residence time of about 7 s per DNA encounter, and can hop from one DNA molecule to another, covering as much as 1 micron (Kad et al., 2010). When UvrA and UvrB were mixed with DNA, a significant percentage of UvrAB (UvrA_2_B or UvrA_2_B_2_) complexes (17%) displayed 1D dimensional DNA sliding and increased residence time to 40 s. Furthermore, analysis of the type of diffusion of the UvrAB complex demonstrated three types of movement on DNA: random diffusion, intermittent motion (in which the protein pauses for several seconds and then proceeds in a random search), and directed motion driven by ATP hydrolysis (Figure 5). In contrast to directed motion, ATP was not required to observe random motion on the DNA. In fact, the number of moving UvrAB molecules increased to 39% in the absence of ATP. Thus, ATP might be required for “clamping down” of UvrB helicase fold at sites of damage and the pausing motion observed may be explained by UvrB entering a closed-down state at potential lesion. By utilizing both 1D and 3D search mechanisms, relatively low numbers (∼40) of UvrAB molecules could efficiently search an entire bacterial genome within the time of one round of bacterial division (∼20 min) (Kad et al., 2010).

Both UvrA and UvrB possess ATPase activity that plays an important role in their search mechanisms. UvrA belongs to the ABC (ATP-binding cassette) ATPase superfamily and has two ATP binding sites per monomer with one near the N and C-termini respectively. In the dimeric state the N-terminal domain of one monomer interacts with the C-terminal domain of the other. Barrett and Kad studied the ATPase activity of UvrA and found that while DNA stimulated UvrA’s ATPase activity 1.4-fold, DNA damage did not alter the steady-state rate of ATP hydrolysis (*k*_cat_), but instead reduced the *K*_m_ for ATP by 3-fold (Barnett and Kad, 2019). Single molecule DNA binding revealed that ATP also increases the time UvrA stays bound to damaged DNA by 3-fold. To explain these interesting results, the authors suggested that UvrA uses negative cooperativity between its two ATP binding sites such that the second ATP site is activated only after UvrA binds DNA damage. By coupling the activity of the second ATPase active site to the binding of damage detection, UvrA can maintain the same steady-state rate of ATPase activity and at the same time increase its binding lifetime.

UvrB contains an autoinhibitory domain which prevents its direct interaction with DNA and inhibits its ATPase activity in the absence of UvrA. UvrB also interacts with UvrC through a c-terminal coiled-coiled domain (DellaVecchia et al., 2007). We, working with Kad and coworkers, found that UvrB binds DNA tightropes in the presence of UvrC, but only if the two proteins are mixed together prior to adding to the DNA (Hughes et al., 2014). About 50% of these UvrBC complexes were motile on the DNA, displaying both random diffusion and anomalous 1D diffusion. Analysis of UvrB mutants affecting the interaction of UvrB with DNA showed that UvrB makes direct contact with the DNA as part of the UvrBC complex. Finally, analysis of an ATP-hydrolysis defective mutant of UvrB compared to WT-UvrB suggested that ATP binding and hydrolysis of ATP changes the conformation of UvrB to induce pausing on the DNA. Thus, ATP hydrolysis by UvrB promotes anomalous diffusion and is consistent with pausing observed with UvrAB complexes (Kad et al., 2010).

The fact that UvrBC complexes can form and migrate on the DNA raises important questions about the potential role of these complexes in the physiological response to DNA damage in bacteria. Expression of UvrC is under tight control in bacterial cells, perhaps due to its dual nuclease activities, but it was discovered that ectopic UvrC expression in a *uvrA* deletion strain increased survival after low to moderate UV-irradiation, but not at high levels (Springall et al., 2018). To better understand the UV protection mechanism of UvrC, Kad and coworkers used oblique angle fluorescence to perform single molecule analysis of purified UvrABC proteins to find that UvrBC complexes recognize lesions almost as efficiently as UvrAB complexes (Springall et al., 2018). Furthermore, UvrABC complexes were observed to migrate together on DNA using three orthogonal Qdot-labeling approaches. Finally, using eGFP-labelled UvrB and UvrC in living cells (also imaged *via* oblique angle fluorescence), they observed UvrBC moving from the cytoplasm to the DNA after UV-damage to *E. coli*. Therefore, upon UV-irradiation, UvrA, UvrB, and UvrC coordinate to efficiently find and repair DNA damage using various searching behaviors mediated by ATP hydrolysis.

### Further Single-Molecule Visualization of Bacterial NER in Live Cells

Studying proteins on DNA tightropes allows for precise localization of binding and measurements of diffusion; however, experiments performed single strand of linear DNA are a simplification of damage detection that occurs within a cell. Instead of one straight piece of DNA (i.e., as a tightrope), damage detection proteins must sift through many loops and coils of genomic DNA, often bound by various other factors (Figure 4). Hence, a growing body of studies has been aimed at understanding how proteins function at the single molecule level within living cells, including recent work showing double-strand DNA break repair in living *E. coli* (Lawaree et al., 2020; Wiktor et al., 2021). Efforts at observing NER at the single molecule scale within living cells have begun with multiple studies imaging prokaryotic NER damage detecting proteins UvrA and UvrB in *E. coli* (Stracy et al., 2016; Springall et al., 2018; Ghodke et al., 2020; Ho et al., 2020). These approaches allow for the visualization of single molecules within cells based limiting the number of fluorophores excited (*via* oblique angle or PALM imaging) bound to the DNA and diffusing slowly give defined fluorescent signal.

As in the DNA tightrope assay, binding lifetimes for DNA repair proteins can be calculated by observing the length of stable signaling. Channeling interactions can be studied by determining the colocalization parameters of two orthogonally labeled proteins. Using near-TIRF microscopy, UvrA in live cells also exhibited multi-exponential cumulative residence time to dissociation (CRTD)–exhibiting one short and one long lifetime of 1.6 and 24 s, respectively (Stracy et al., 2016; Springall et al., 2018; Ghodke et al., 2020). These values agree with previously published lifetimes using purified UvrA, in which a single lifetime of ∼7 s was observed. Upon UV-irradiation of the cells, the long lifetime increased but the short lifetime did not change. These results indicate that, even though the exact positions of the DNA damage may not be known within the cells, that the long-lived (24 s) events may represent UvrA bound to damage sites.

This live-cell imaging approach has also been applied to NER proteins repairing damage aside from just UV-damage. For example, the cellular response to the toxin DarT that ADP-ribosylates ssDNA, and in turn reduces cell growth by limiting replication, has been studied at the single molecule level (Lawaree et al., 2020). When ADP-ribosylation of ssDNA cannot be reversed by the protein DarG, the modified DNA is processed first by homologous recombination pathway to convert the ssDNA to dsDNA. Following this conversion, NER removes the damaged substrate and replaces it with undamaged DNA. Using photoactivatable mCherry for PALM, live-cell single-molecule imaging was able to capture changes in UvrB diffusivity upon induction of DNA damage by overexpression of DarT (Lawaree et al., 2020). On examining the diffusivity of single UvrB molecules, expression of active DarT increased the proportion of immobile UvrB (average diffusivity less than μm^2^ x s^−1^) by fourfold via a decrease in the population with faster diffusion. Thus, this single-molecule study in live *E. coli* revealed UvrB searching for and detecting a new form of damage in real time.

### Single-Molecule Studies of Mammalian NER Proteins: UV-DDB

**FIGURE 6:**
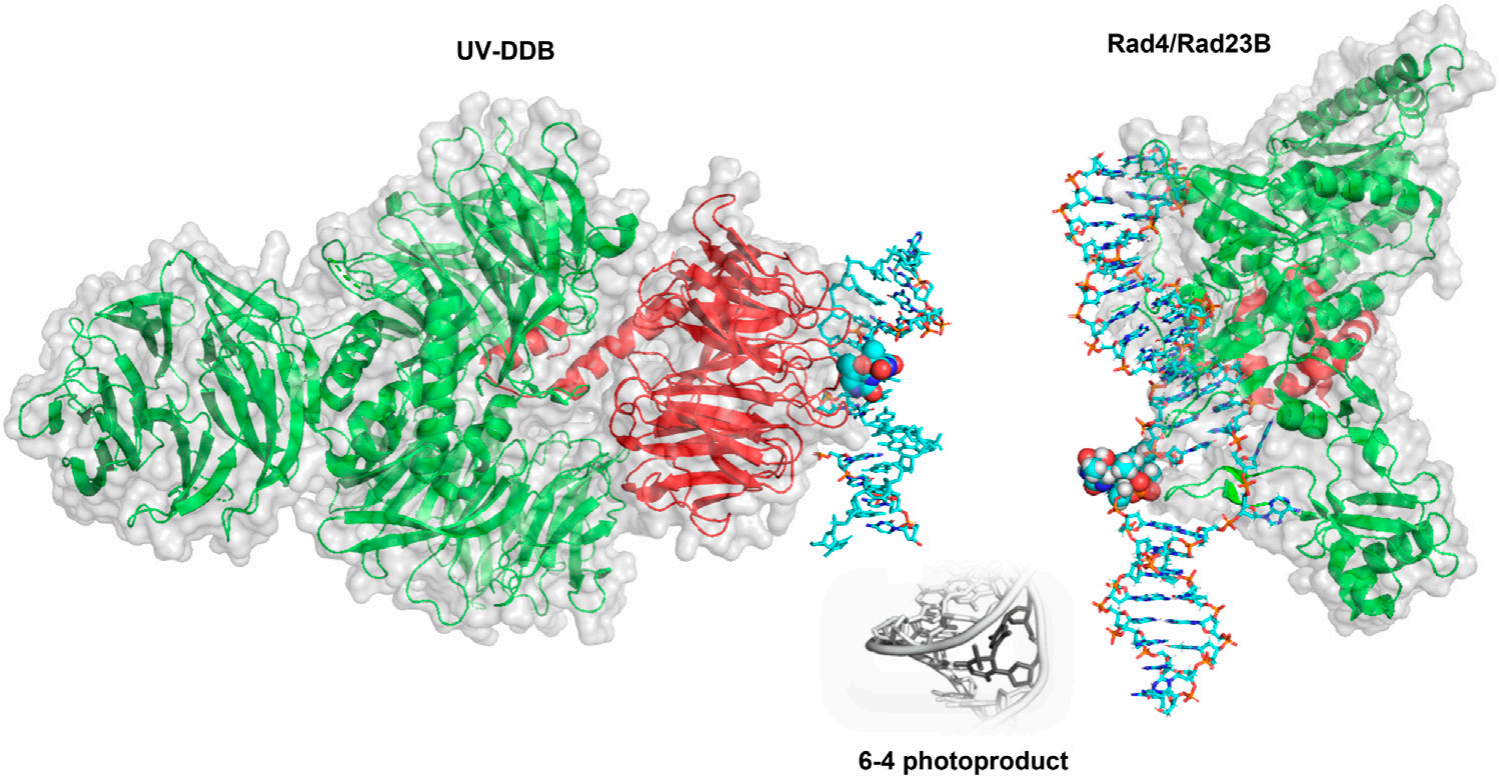
Crystal structures of UV-DDB and Rad4/Rad23 bound to damaged DNA. **(Left)** DDB1 (green cartoon) and DDB2 (red cartoon) form a UV-DDB heterodimer. In this structure, UV-DDB is bound to DNA (cyan sticks) with a 6-4 photoproduct and adjacent base flipped into the recognition domain (spheres colored by atom). Structure taken from PDB ID: 3EI1. **(Right). **Crystal structure of Rad4/Rad23 (green and red cartoons, respectively) bound to a UV-induced 6-4 photoproduct containing DNA (cyan sticks). The 6-4 photoproduct is displayed as spheres. Structure taken from PDB ID: 6CFI. While it is widely believed to occur, the direct hand-off of a 6-4 photoproduct from UV-DDB to XPC has not been directly observed by single molecule techniques.

In mammalian cells, the recognition protein responsible for initiating detection of DNA products in chromatin is UV-DDB (see Figure 6 for structure). Understanding how UV-DDB undergoes damage searching was uncovered by analyzing Qdot labeled UV-DDB binding undamaged DNA tightropes as well as tightropes damaged by UV radiation, visualized with oblique angle fluorescence microscopy (Ghodke et al., 2014). By assaying hundreds of single-molecule binding events, the CRTD could be fit to a triple-exponential function with three lifetimes: including one at 0.3–0.8 s, one at 8.1 s, and one at 113–126 s. These events that vary over several orders of magnitude were each hypothesized to represent a cascade of recognition steps called conformational proofreading in which both UV-DDB and DNA undergo discrete structural changes to increase overall binding affinities and therefore longer dwell times. Importantly, a bulk solution measurement of UV-DDB binding DNA would not allow for each of these states to be characterized because all events would be averaged together in an ensemble measurement.

Different binding distributions were observed for the damaged and undamaged tightropes, particularly in the number of persistent DNA binding events (binding events that lasted greater than 900 s). The damaged DNA substrate exhibited over a two-fold increase in the proportion of persistent binding events, supporting a model that once UV-DDB locates a damage site it halts its search and uses conformational proofreading to achieve tight binding to sites of damage. Based on previous information about UV-DDB dimerization, two different Qdots were conjugated UV-DDB to test if multiple molecules of UV-DDB made up one persistent binding event. Colocalization was observed the two Qdots, which, along with previous atomic force microscopy and electron microscopy data, implies that UV-DDB dimerization occurs at some persistent binding events and may play an important role in damage recognition by UV-DDB (Yeh et al., 2012). This study also showed that the K244E variant of DDB2 responsible for xeroderma pigmentosum complementation group E (XPE) displayed sliding on DNA and was unable to stably bind to damaged sites. Finally, WT UV-DDB protein was found to undergo anomalous diffusion when Mg^2+^ is supplied in the binding buffer (Jang et al., 2019; Beecher et al., 2020). This diffusion apparently increases the specificity window of UV-DDB for a wide range of lesions including 8-oxoG (Jang et al., 2019; Beecher et al., 2020).

### Damage Identification by XPC-RAD23B

Single-molecule studies also revealed important details about damage detection by XPC-RAD23B (Figure 6), another NER protein involved in DNA damage detection. The yeast homolog to this complex (Rad4-Rad23) was studied by oblique angle fluorescence microscopy *via* the DNA tightrope assay as it searched for UV-photoproducts along UV-irradiated lambda DNA at the single molecule scale (Kong et al., 2016). In a similar manner to that of the UV-DDB, this experimental setup allowed for the characterization of individual complexes–therefore, many more details could be uncovered about the search mechanism than if all the behavior was averaged together in bulk solution. The search behavior could be separated into three different groups: on DNA with 20 J/m^2^ of damage, ∼60% of events were nonmotile (stably bound to the DNA), ∼25% exhibited random diffusion (moving across large amounts of DNA of at least several thousand bp), and ∼15% showed anomalous diffusion (moving back and forth across ∼500–1,000 bp of DNA). The distributions of molecules exhibiting each type of search changed depending on the lesion used, revealing another layer of the complexities of the XPC-RAD23B search mechanism (Kong et al., 2016).

**FIGURE 7:**
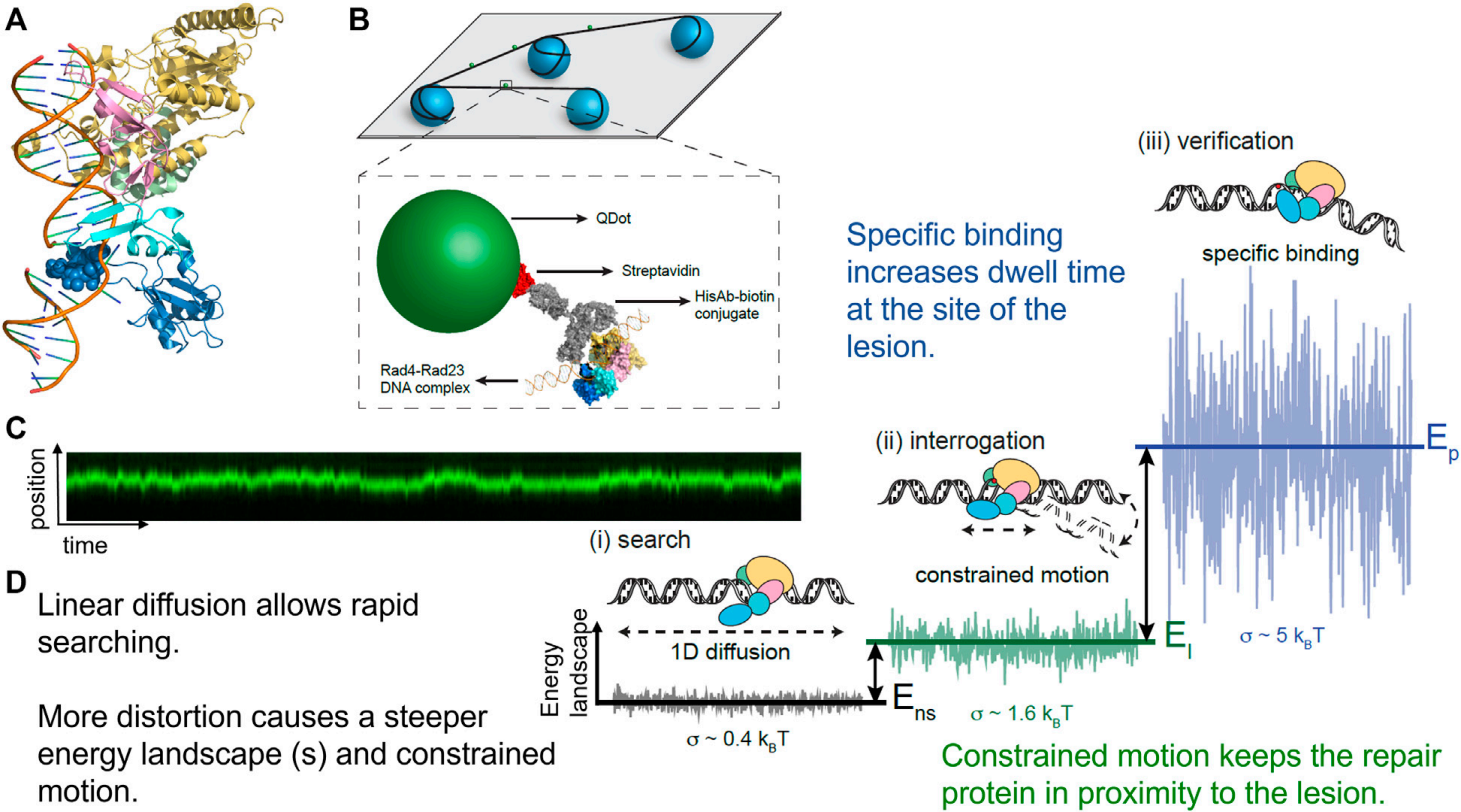
Rad4-Rad23 displays anomalous diffusion on DNA tightropes around UV-induced photoproducts. **(A)** Co-crystal structure of Rad4-Rad23 bound to DNA containing a CPD-mismatch. PDB ID: 2QSG (Min and Pavletich, 2007). **(B)** DNA tightrope assay and Qdot-protein conjugation strategy. **(C)** Example of a kymograph showing binding position on the Y-axis and time on the X-axis of a Rad4-Rad23 particle displaying anomalous diffusion/constrained motion. This particle moved between 500–1,000 bp around a cyclobutane pyrimidine dimer. **(D)** Depiction of how changes in protein-DNA conformation with closing down of beta-harpin 3 in dark blue alter the diffusivity of a particle by creating a steeper energy landscape and in turn lead to reduced linear diffusion, constrained motion, and finally a non-motile specific DNA complex. Together these changes in protein-DNA conformation help solve the “speed-stability” paradox of allowing rapid diffusion along DNA for lesions, and specific damage detection by DNA repair proteins such as Rad4, XPC, and PARP1. Figure adapted with permission from (Kong et al., 2016).

By engineering a cyclobutane pyrimidine TT dimer into a 2 kb plasmid that was ligated together to form a CPD array, it was found that Rad4-Rad23 performed anomalous diffusion around the CPD (Figure 7). Thus, these data helped to explain the odd paradox that Rad4-Rad23 is essential for CPD removal in yeast, but that purified Rad4-Rad23 does not bind specifically to a short segment of DNA containing CPD. This anomalous diffusion phenomenon causes Rad4-Rad23 to simply slide off of short segments of DNA in biochemical assays and thus appear as if no recognition is achieved. This concept of anomalous diffusion also helps solve a potential traffic jam of repair factors binding to the same site, which might cause steric interference at the lesion (Kong and Van Houten, 2017). Finally, using AFM we were able to show that Rad4-Rad23 bound to a lesion bent the DNA by 43°, and that the third beta-hairpin binding motif which flips out the damaged bases, was not necessary for DNA bending. More recently, similar findings about the heterogeneous search mechanisms for the human Rad4 homolog, XPC-RAD23B, suggesting a conserved search mechanism between species (Mu et al., 2018; Cheon et al., 2019). Furthermore, by including “road-block” proteins to prevent DNA sliding, insights could be gained that XPC-RAD23B utilizes a hopping mechanism to survey DNA, rather than solely sliding in constant contact as it searches for damage.

### XPA Detects Damage on DNA Tightropes

Single-molecule approaches have also proven invaluable for studying damage search and recognition mechanisms of XPA, another essential NER protein with multiple roles as both a damage sensor and scaffolding protein (Beckwitt et al., 2020). In a similar approach to other studies of NER proteins, XPA was conjugated to a Qdot and the mechanism of its search revealed as it scanned along DNA tightropes both with and without UV damage using oblique angle fluorescence microscopy. By parsing the events down to the single molecule level, it was revealed that XPA’s search behavior could also be classified into stationary binding events and binding events that showed motion along the DNA, including short-range (between 130–690 nm or ∼380–2000 bp of displacement) motion and long-range (>690 nm) motion. Upon the induction of DNA damage, the number of binding events without motion increased and the number of events that exhibited motion decreased, particularly the long-range motion events. Furthermore, XPA displayed episodic pausing of motion on DNA. These discontinuous sliding events were both dependent upon damage and large disordered regions of XPA on the N- and C-termini of the protein. Using AFM imaging we also found that XPA bends the DNA by about 60° during each binding event. We hypothesize that once the XPA protein fully engages with a damage site, the disordered regions fold down on DNA and increase binding energy to the point that thermal motion can no longer drive XPA along the DNA, thereby pausing its motion. Conversely, unfolding of these disordered arms allows XPA to begin sliding. Hence, the presence of DNA damage changes the search behavior of XPA, altering the behavior from surveying long stretches of undamaged DNA to stationary binding or facilitated diffusion around positions with damage present.

### TFIIH Subunits p44/p62 Sensing Damage

In both GG-NER and TC-NER, the final verification step is achieved by the action of TFIIH, which binds avidly to damage sites and opens up a bubble around the damage of approximately 25 bases. TFIIH consists of 10 subunits, including XPD and XPB, proteins that provide DNA translocation and helicase opening of the DNA, respectively, as well as the three subunit CAK complex. The CAK complex is known to dissociate during damage verification by XPD, leaving the remaining core structural subunits are p62, p52, p44, p34, and p8. XPD displays a very poor helicase activity unless bound by p44, reviewed in (Kuper and Kisker, 2021). The Kisker and Kad groups have recently collaborated to investigate how single molecules of p44/p62 interact with non-damaged and UV-irradiated DNA without XPD or XPB using oblique angle fluorescence microscopy (Barnett et al., 2020). With undamaged DNA, the p44/p66 complex diffuses on DNA with ∼80% of the observed molecules showing motility. Surprisingly, the introduction of large amounts of UV-damage to the DNA reduced the number of motile molecules down to 50%. Furthermore, many of the motile molecules on UV-irradiated DNA displayed constrained motion resembling anomalous diffusion. Since this complex binds more avidly to DNA containing single-strand/double-strand junctions than to duplex dsDNA, the authors suggested that perhaps p44/p62 complex is recognizing transient opening of the helix induced by UV-induced photoproducts. Thus, even proteins involved in the later steps of NER may also contribute to proper differentiation of damaged DNA from undamaged DNA.

### Single-Molecule Characterization of DNA Glycosylases

**FIGURE 8:**
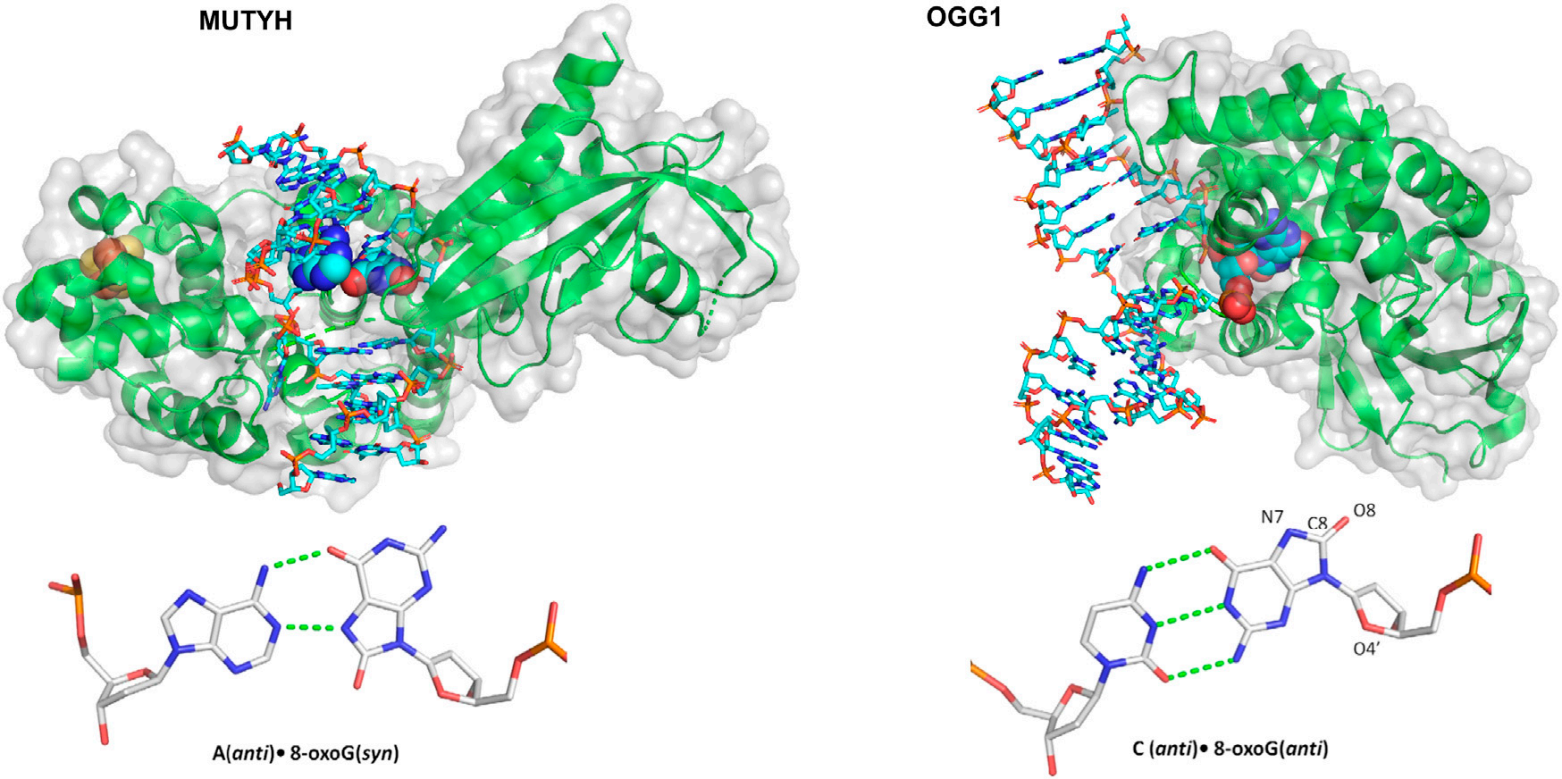
Structures of two mammalian glycosylases bound to their damaged substrates. **(Left)** Crystal structure of MUTYH (green ribbons) bound to DNA (cyan sticks), containing the oxidative damage substrate A:8-oxoG (displayed as spheres). Its Iron-sulfur cluster is displayed as orange and yellow spheres. Structure taken from PDB ID: 4YPH. **(Right)** OGG1 crystal structure (green ribbons) bound to a damaged DNA (cyan sticks) substrate containing a C:8-oxoG base pair (spheres). Structure taken from PDB ID:1EBM.

One landmark study in understanding how glycosylases detect their damage was published by the Verdine group, using a single molecule approach similar to the DNA tightrope approaches discussed in the NER section (Blainey et al., 2006). Instead of tightropes strung between beads, however, one end of *λ* DNA was anchored to the bottom of a slide with a biotin-streptavidin linkage, followed by an establishment of flow to extend the DNA. Then, the interaction of Cy3-labeled 8-oxoguanine glycosylase 1 (OGG1) (Figure 8) with undamaged DNA could be studied at the single-molecule level using TIRF microscopy. Both the binding lifetime and the diffusivity of the protein was assayed; these processes were shown to be salt concentration dependent as well as pH dependent. The average diffusivity of OGG1 on undamaged DNA was ∼0.5 μm^2^ per second under physiological conditions. However, further analysis of the data revealed that the diffusion of OGG1 was better modeled by a two-state model: one state with fast diffusion and one state with slower diffusion, potentially generated by two physical conformations of OGG1 bound to the DNA. Using both of these states together, OGG1 can sample much of the DNA for damage sites while balancing the need to survey long stretches of DNA (Vestergaard et al., 2018).

Another step forward in mechanistic understanding of how glycosylases search for and repair oxidative damage came from the Wallace laboratory, who examined three different bacterial glycosylases: endonuclease III (Nth), endonuclease VIII (Nei), and formamidopyrimidine DNA glycosylase (Fpg) (Nelson et al., 2014). All three of these glycosylases remove various types of oxidative damage–to generate damaged DNA with the appropriate target substrate, DNA was treated with either osmium tetroxide and heat (to form thymine glycol) or methylene blue plus visible light (to form 8-oxoG). The damaged DNA was suspended between beads to form a tightrope, and then Qdot-labeled glycosylases flowed in to observe how they detect their damage *via* oblique angle fluorescence. As with OGG1, the binding events exhibit diffusivity that varied over several orders of magnitude. As the damage increased, however, the binding lifetimes increased and average diffusivity decreased, presumably as the proteins identified and bound the damage.

Active site mutants were generated to eliminate the glycosylase wedge (a hydrophobic residue that inserts into the DNA helix after the base is flipped out), including F111A, Y72A, and L81A for Fpg, Nei, and Nth, respectively. For these wedge mutants, average diffusivity increased to even greater than the WT levels and did not reduce as much in the presence of damage. In other words, mutating each glycosylase wedge residue prevented the glycosylases from finding their target lesions. Instead, they diffused more rapidly, passing by their substrates without binding (Nelson et al., 2014). More recently, the wedge residue of MUTYH, a glycosylase, Figure 8, that binds 8-oxoG across from A and excises the A, was studied with similar single molecule approaches, this time with near-TIRF microscopy. Similar to bacterial glycosylases, this MUTYH variant (Y150C) showed one state diffusivity and did not show a diminished rate of diffusion on tightropes containing its 8-oxoG:A substrate (Nelson et al., 2019). Importantly, this homologous mutation has clinical significance and is associated with MUTYH-associated polyposis, suggesting that the defects in damage detection contribute to the disease. Several other mammalian DNA glycosylases and other BER proteins have not been studied using DNA tightropes, but sophisticated enzymology and other single-molecule approaches have revealed much of their mechanism. For instance, the unique non-base flipping glycosylase AlkD search mechanism was studied on the single molecule level with a Förster resonance energy transfer (FRET)–fluorescence correlation spectroscopy (FCS) approach (Peng et al., 2020). Finally, it should be noted that innovative kinetic regimes involving varying lengths of DNA substrates to visualize processivity has improved the understanding of how BER proteins search for damage including alkyladenine DNA glycosylase, uracil DNA glycosylase, and even other downstream BER enzymes (Hedglin and O'Brien, 2008; Porecha and Stivers, 2008).

### Visualizing Damage Detection and Handoff by Downstream BER Enzymes

**FIGURE 9:**
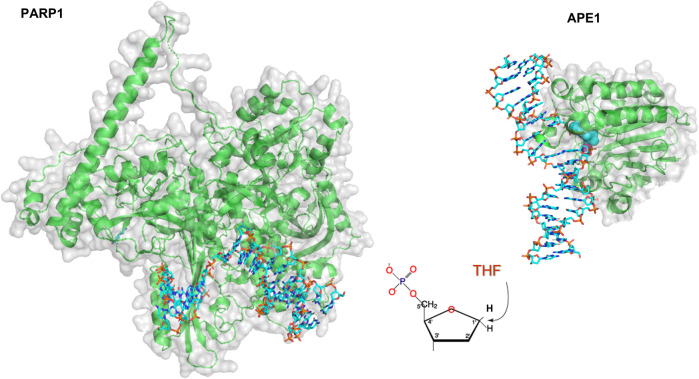
Models of PARP1 and APE1 bound to DNA repair intermediates. **(Left)** PARP1 (green cartoon) bound to DNA (cyan sticks) containing a single strand break. Structure was created using Alpha-fold generated structure (Jumper et al., 2021) and docking in the DNA from a co-crystal structure of Pol β bound to a nicked DNA substrate (PDB ID: 4KLO) using HDOCK (Yan et al., 2020). **(Right)** Crystal structure of APE1 (green cartoon) bound to DNA (cyan sticks) containing an abasic site analog, THF. APE1 taken from PDB code 5DFF.

The number of single-molecule studies for downstream BER enzymes binding their substrates is more limited than those of glycosylases. Using an innovative kinetic approach Wilson and colleagues showed that DNA Pol *ß* lyase activity can act processively, in a hopping manner if the lesions are within a mean distance of 24 bp (Howard et al., 2017). In collaboration with the Wilson laboratory, we used oblique angle fluorescence microscopy to perform single-molecule studies of two other mammalian BER enzymes, PARP1 and APE1, simultaneously searching and binding for their substrates embedded in DNA tightropes (AP sites). PARP1 has been shown to bind avidly to abasic sites, but this binding does not activate its parylation activity. By visualizing this search at single molecule scale, it was found that PARP1 undergoes 3D diffusion to identify its substrates; that is, its diffusion mostly occurred within solution, not along the DNA, so PARP1 bound to DNA exhibited a stable position (see Figures 4, 9). However, the behavior of PARP1 changed in the presence of APE1: instead of stable binding events from 3D diffusion alone, PARP1 also exhibited 1D diffusion along the DNA and shorter binding lifetimes (Liu et al., 2017). This behavior is consistent with APE1 facilitating the dissociation of PARP1 from the substrate. The search behavior of PARP1 could also be altered depending on its post-translationally modifications (PTM). When PARP1 was auto-PARylated, it diffused much more rapidly and exhibited 1D diffusion even without APE1 present. Furthermore, when both enzymes were viewed simultaneously on DNA, the way that they cooperate to facilitate repair was clarified. The two enzymes colocalized on 6% of the events from tightropes containing AP sites cleaved by APE1, suggesting a high degree of cooperation between the proteins. Altogether, many creative single-molecule approaches have led to an increased understanding of how BER enzymes detect and repair their substrates.

Similar to NER, new imaging techniques have enabled single-molecule level imaging for BER enzymes in living cells. While no studies of this nature have been performed with upstream BER enzymes to our knowledge, both prokaryotic Pol I and DNA ligase were studied using PALM at the single molecule scale in live *E. coli* (Uphoff et al., 2013). In this work, the endogenous proteins were replaced by versions tagged with a photoactivatable mCherry, enabling only a small subset of the fluorescent proteins to be excited at a time. By introducing alkylative damage with methyl methanesulfonate (MMS), differences in the search behavior at the single molecule scale could be monitored. Of these single-molecule events, diffusivity was measured, with populations shifting exhibiting average diffusivities of ∼0.8 μm^2^·s^−1^ for Pol I and ∼1 μm^2^·s^−1^ for ligase. Using this technique, molecules bound to DNA exhibited much slower than other molecules in the nucleoid with diffusivity of ∼0. Of note, these are the populations that would be assayed with the tightrope assay, and the average values are typically 3 orders of magnitude lower than the values for unbound molecules in the nucleoid. The average diffusivity of both Pol I and DNA ligase decreased upon DNA damage, with the percentage of particles bound shifting ∼5-fold from <5 to 13% or 18%, respectively. In contrast, the diffusivity of Fis, a DNA binding protein without DNA repair activity, did not significantly alter upon MMS treatment, furthering the case that the very slowly diffusing molecules were DNA repair proteins bound to damage sites in the genome. Using these details, the authors were able to generate a complex model of downstream BER, in which induction of DNA damaged dramatically increased the proportion of bound Pol I and ligase molecules, reducing their search time by ∼6 fold to respond to MMS treatment.

### Crosstalk Between BER and NER

NER and BER proteins canonically have their own sets of lesions as outlined previously, with NER responsible for repairing bulkier adducts, such as those formed by UV-damage, and BER enzymes detecting individual base damage created by oxidation and alkylation. Both pathways, however, share a similar task of searching through billions of nucleotides for DNA damage to guard genomic stability. As more has been uncovered about BER and NER, a growing body of literature points to the importance of crosstalk between the pathways. In particular, the role of NER proteins in the repair of oxidative DNA damage has begun to emerge (Reardon et al., 1997; D'Errico et al., 2006; Parlanti et al., 2012; Limpose et al., 2017). These interaction networks are complex, and the exact biological importance of pathway crosstalk is still under investigation. However, various proteins in both pathways are involved in the crosstalk between pathways, including the NER proteins UV-DDB, XPC-RAD23B, XPA, CSB and XPG, and BER enzymes OGG1, NTHL1, APE1, and MUTYH (Kumar et al., 2020; Jang et al., 2021). Using single-molecule fluorescence assays, direct molecular evidence for the importance of how pathway crosstalk contributes to the detection of DNA damage has recently begun to emerge.

Utilizing a DNA tightrope to watch these two pathways interact together in real time, more details about the mechanism by which UV-DDB coordinates with mammalian BER enzymes OGG1 and APE1 were revealed (Jang et al., 2019). By using oblique angle fluorescence to visualize the search and binding of Qdot labeled OGG1 and APE1 on tightropes containing abasic sites (the product of OGG1 and the substrate for APE1), it was found that the addition of unlabeled UV-DDB, even at 10-fold excess, did not greatly alter the motility of either enzyme. This result implies that if unlabeled UV-DDB was stably bound to the DNA tightrope it did not act as a roadblock for the damage detection of BER enzymes. However, the presence of UV-DDB greatly reduced the binding lifetime of both enzymes, facilitating the dissociation of both APE1 and OGG1 from abasic sites. Therefore, UV-DDB apparently plays an important role of facilitated dissociation of both OGG1 and APE1 allowing turnover of these enzymes. In another experimental regime, UV-DDB and OGG1 or UV-DDB and APE1 were simultaneously labeled with two colors of Qdots and studied as both enzymes searched for abasic sites along a DNA tightrope. Interestingly, a number of these events showed colocalization between the two enzymes: nearly 10% of all events in both cases. Of these colocalized events, some represented events where both proteins stably bound at a damage site, and some represented events where the two proteins would diffuse together. Thus, direct evidence was observed that UV-DDB cooperates with BER enzymes to detect DNA damage. In the same study, a unique chemoptogenetic approach was used to introduce 8-oxoG to telomeres of cells, and UV-DDB rapidly bound these damage sites, with recruitment even more rapid than OGG1, the glycosylase responsible for their repair (Jang et al., 2019). Potentially, this combined search process could be UV-DDB assisting BER enzymes to find their lesion in the context of chromatin as well as to work to dissociate the enzyme-product complex.

The crosstalk between UV-DDB and another mammalian glycosylase, MUTYH, was also recently studied by our group working in collaboration with the David laboratory at the single-molecule level (Jang et al., 2021). UV-DDB was shown to stimulate the enzymatic turnover of MUTYH and facilitate the dissociation of MUTYH *via* transient co-complex formation. Strikingly, when compared to APE1 (the next step in BER), much lower concentrations of UV-DDB (∼50-fold less) were required for MUTYH to dissociate from its product or form a co-complex–the co-complex observed *via* electrophoretic mobility shift assay (EMSA) was confirmed with atomic force microscopy, in which volumes measured were consistent with UV-DDB-MUTYH co-complexes on DNA. Hence, single-molecule approaches were employed to better understand the mechanism by which these two proteins work together. In a DNA tightrope approach similar to that performed with OGG1 and UV-DDB, the two orthogonally labeled proteins were studied as they searched for damage on abasic-site containing DNA tightropes with oblique angle fluorescence. Out of 200 events observed, 24% consisted of colocalized MUTYH and UV-DDB. Furthermore, including UV-DDB in the assay reduced the binding half-life of MUTYH 15-fold, further supporting a model in which UV-DDB increases the rate of MUTYH dissociation from its product. Therefore, UV-DDB was shown to cooperate with yet another BER glycosylase to detect and repair DNA damage, further solidifying the multifunctional role that it plays in both BER and NER.

## Conclusion

Damage detection systems from both BER and NER pathways harness the architecture of the DNA itself to better interrogate for damage, transitioning from a 3D diffusion search mechanism to a mechanism that involves diffusing along the DNA in a one-dimensional fashion, hopping and/or sliding down the double helix. Another common feature between NER and BER damage detection is that damage detection proteins need to detect their substrates and bind them, but also need to eventually hand them off to downstream proteins in the pathway. In both pathways, proteins have been observed to undergo constrained motion on damaged DNA (including PARP1, XPC-RAD23B, XPA, p44/p62 and UV-DDB), oscillating back and forth over short ranges of DNA, potentially allowing other factors to be recruited for the repair (Liu et al., 2017; Cheon et al., 2019; Jang et al., 2019; Barnett et al., 2020; Beckwitt et al., 2020). Interestingly, anomalous diffusion has been observed less frequently with DNA glycosylases. One potential reason why less anomalous diffusion is observed is that glycosylases cleave the DNA once the damage has been detected, creating a fragile abasic site or potentially nicked DNA behind, so many glycosylases exhibit high affinity for their products as well as their substrates. Therefore, instead of undergoing anomalous diffusion, some glycosylases may remain tightly bound to their products as a way of protecting these potentially harmful repair intermediates. Because glycosylases undergo so many different mechanisms, it is also possible that there are some also undergo a combination of anomalous diffusion and/or tight binding of their product that may be uncovered in the future.

### Outlook

While single molecule studies have advanced our understanding of some glycosylases, many others have not been interrogated at this level and thus remain to be studied. Most effort so far has been dedicated to the enzymes that initiate the repair pathways, but the way that downstream multiple proteins find their interaction partners, and facilitate efficient hand-offs is not known. Furthermore, it was already shown that PTMs on PARP1 drastically changed its search mechanism–future studies may show how PTMs alter other damage detection enzymes. Although several live-cell studies have been performed at single-molecule scale with NER proteins, there has thus far been minimal live-cell single-molecule studies published on BER proteins, particularly for the glycosylase step of BER. Future applications of superresolution microscopy will lead to a better understanding of how BER functions in a live-cell context. As imaging techniques improve, visualization of both pathways at single molecule scale in living mammalian cells may become possible, enabling the study of these pathways at an even more relevant context. If that were attainable, it would be fascinating to see how the behavior changes in a live cell upon the addition of a chemotherapeutic or DNA repair inhibitor.

Another question facing the field is the role that DNA bending plays in damage detection. Many structures and studies with electron microscopy and atomic force microscopy have revealed that damage detection enzymes impart a significant bend on the DNA, in some cases up to 90° (or more) from canonical B-form DNA (see Figure 3) (Beckwitt et al., 2018). The exact role that DNA bending plays on damage search and detection remains unknown, however. Technical limitations have so far prevented DNA bending to be studied simultaneously with the DNA search process (i.e., while performing a DNA tightrope assay, where all the DNA tightropes are suspended at similar tensions created by the flow cell). As more user-friendly technology emerges, such as the Lumicks C-trap^®^ combining optical tweezer/force measurements with confocal microscopy and microfluidics, DNA tension can be measured and altered in real-time during the search process (Rill et al., 2020; Belan et al., 2021); for instance, applying high tensions would prevent DNA bending and potentially alter search behavior. Using optical tweezers fluorescence microscopy approach, DNA tension was previously demonstrated to induce Cas9 off-target activity, so it will likely also influence DNA damage search and recognition (Newton et al., 2019). The future holds great promise for the convergence of single molecule analysis of purified proteins with super-resolution approaches in living cells (Boden et al., 2021) to better describe how DNA repair proteins solve the enigmatic problem of finding rare lesions in a sea of non-damaged DNA.

## Glossary

8-oxoG: 8-oxoguanine
ABC: ATP-binding cassette
AFM: atomic force microscopy
AlkD: alkylpurine glycosylase D
APE1: apurinic/apyrimidinic endonuclease 1
ATP: adenosine triphosphate
BER: base excision repair
CAK: CDK-activating kinase
CENT2: centrin 2
CPD,: cylcobutane pyrimidine dimer
CRTD: cumulative residence time to dissociation
CSA: Cockayne syndrome type A protein
CSB: Cockayne syndrome type B protein
EMSA: electrophoretic mobility shift assay
ERCC1: excision repair cross complementing group 1
εA: 1,N6-ethenoadenine
FapyG: 2,6-diamino-4-oxo-5-formamidopyrimidine
FEN1: flap endonuclease 1
Fpg: formamidopyrimidine DNA glycosylase
HILO: highly inclined and laminated optical sheet
Nei: endonuclease VIII
NER: nucleotide excision repair
Nth: endonuclease III
NTHL1: endonuclease III Like DNA Glycosylase 1
OGG1: 8-oxoguanine glycosylase 1
PALM: photoactivated localization microscopy
PARP1: Poly [ADP-ribose] polymerase 1
PDB: protein data bank
PNK: polynucleotide kinase
Pol β: DNA polymerase β
PTM: post-translational modification
Qdot: quantum dot
ROS: reactive oxygen species
STORM: stochastic optical reconstruction microscopy
TFIIH: transcription factor IIH
TIRF: total internal reflection fluorescence
UDG: uracil DNA glycosylase
UV-DDB: UV-damaged DNA-binding protein
UvrA: UvrABC system protein A
UvrB: UvrABC system protein B
UvrC: UvrABC system protein C
UvrD: DNA helicase II
XPA: xeroderma pigmentosum complementation group A protein
XPB: xeroderma pigmentosum complementation group B protein
XPC: xeroderma pigmentosum complementation group C protein
XPD: xeroderma pigmentosum complementation group D protein
XPE: xeroderma pigmentosum complementation group E protein
XPF: xeroderma pigmentosum complementation group F protein
XPG: xeroderma pigmentosum complementation group G protein
XRCC1: X-ray repair cross-complementing protein 1
